# Some Observations on the Prevailing Opinions on the Modus Operandi of the Causes Producing Compression of the Brain

**Published:** 1820-09

**Authors:** 


FOR THE LONDON MEDICAL AND PHYSICAL JOURNAL.

### *Some Observations on the prevailing Opinions on the Modus Operandi of the Causes producing Compression of the Brain.*

THE object of the following detail, is to expose to view some incongruities which appear to the writer to exist in the reasoning of medical men on the subject of the *modus operandi* of the causes which produce those symptoms generally attributed to a compression of the brain. It will be needless here to give a detail of such symptoms, as they are well known to all who have had any experience in surgery.

It is asserted that the brain, from its very nature and situation, is incompressible: this, Mr. C. Bell, in his Lectures, informs us, was demonstrated by Professor Monro in the following manner: he took as much brain as exactly filled a vessel, (a pint for instance,) and endeavoured to compress it into a less space, but he found it impossible; and hence concluded the same impossibility to obtain when the brain is in the cranium, which, it is presumed, it completely fills during life, when the blood, of course, keeps its vessels in a manner filled, but which, when the brain was in the vessel, would be the reverse, (collapsed.) The inference drawn from this experiment was this, viz. that, when a part of the cranium is depressed, or when there is an extravasation of blood, or an effusion of serum, &c. within it, the brain itself does not suffer compression, but the blood-vessels do; and, in proportion to the bulk of the compressing body, will the capacity for the internal circulation of the head be diminished: and Mr. Bell further asserts, that the symptoms arising from the pressure are the effects of this exclusion of blood from within the head, and not from the compressing body being in contact with, and perhaps irritating, (as he calls it,) or otherwise discomposing, the brain; and since, says he, a proper supply of blood to this organ, which requires nearly five times as much as any other in the body, is so essential to the performance of its functions, a slight diminution of that supply must more or less derange or impede it in this respect: and to this are the symptoms to be attributed. In support of such doctrine, he adverts to syncope as a plain proof: here, says he, there is nothing compressing the brain, and yet the symptoms are nearly the same. He goes on, and says, Lay the patient in the horizontal position, and sensibility will return in half the time it would do if the person remained in the erect position; because the brain, in the horizontal, receives a greater and quicker supply of blood, than in the erect position.

Some, indeed, have gone so far in this theory as to imagine that the symptoms in a case of compression are proportionate to the diminished capacity for the internal circulation, never taking into account the injury which the brain may have suffered in its structure, from the undue pressure which must generally occur more or less, according to the situation, bulk, form, and density, of the body compressing. If the inner table of the cranium be driven in to any considerable depth, the structure of the brain must undoubtedly suffer; but, if only blood be extravasated, it being first in a fluid state, and poured out gradually, the brain will be capable of resistance to a certain extent, and will adapt itself to the form of the compressing body, without suffering any lesion of structure.

It is, I think, pretty evident, that those who pursue this mode of reasoning, hold the brain to be an organ endowed with little sensibility, or indeed vitality; for one would be led to conclude, from such arguments as the foregoing, that the substance of the brain was a senseless mass, and that the blood which circulated through it was every thing: nay, in short, we might say, take away or injure the brain as you will, leave but the blood-vessels free, and there will be no symptoms.

In support of this doctrine, it is usual to appeal to the cases where part of the brain has been lost, and the patients have recovered, as if no such loss had been suffered.

The symptoms of phrenitis and hydrocephalus acutus have also been advanced in support of this doctrine, by referring the increased sensibility which is apparent in the first stage of these complaints, to the quickened action and increased flow of blood to the brain, by which its functions are for a time accelerated or improved; but, when the capacity for the circulation within the cranium is diminished by the subsequent occurrence of effusion, then are its functions proportionately impeded, and at length, as the effusion increases, are altogether suspended, and death is the result.

Now, I believe, the generality of medical men take a different view of the subject, and never once take into consideration the circumstance of there being a diminished capacity for the circulation proportionate to the degree of pressure, but attribute all the symptoms to the irritation, as they call it, arising from the compressing body being in contact with, or pressing upon, the nervous matter of the brain, so as to discompose or alter the relative situation of its minute parts, and thus cause a suspension of some, or a derangement of its whole functions; and hence, say they, arise the many singular symptoms, as a paralysis of one or more of the extremities, or of a particular class of muscles; a partial or total loss of any particular sense, as that of smell, taste, hearing, &c. which sometimes occurs after either a compression or a concussion: in which latter case we may presume there has been an alteration in the relative position of some of its parts, where the nerves supplying those organs arise.

Neither of these arguments are at all satisfactory: there is, I think, something connected with this affection totally inexplicable, since the causes which are found to produce such symptoms as those which are generally attributed to a compression of the brain, do very frequently essentially differ from what is in reality a compression in their very nature, and we must also conclude, in their mode of action: for instance, if a small quantity of matter, say a drachm, secreted between the cranium and dura mater, is known to produce the very same train of symptoms as when the skull has been driven in upon the brain, or when an extravasation of blood to ten times the bulk of the matter has taken place. Now, it would be monstrous to attribute the symptoms produced by this drachm of matter to a diminished supply of blood within the head, in consequence of its bulk or pressure; because there is, in fact, generally, an increased flow of blood to, and a quickened action of the vessels within, the cranium, connected with a febrile state of the system, concomitant with, and dependant upon, the disease which produced the secretion of the matter.

Now, what I have to say on the doctrine of the incompressibility of the brain, is that the term compression is taken in some other than the commonly-accepted sense, or that those who contend for the incompressibility of the brain deny that the nervous matter of the brain is endowed either with sensibility or vitality.

What I have to observe respecting the doctrine of irritation, is, that the facts and arguments adduced in support of it, or something else which it is intended the term irritation should convey, are more substantial and consonant with the theory of the nervous system than any I am acquainted with; but, notwithstanding, they are totally unsatisfactory.

The term irritation is here very much misapplied, since we have not a single symptom of irritation; unless, merely for the sake of keeping the term, we say it is quite a different kind of irritation, and manifests itself by a total loss of sensibility: but who, I would ask, in their senses, will apply the same term to two affections as manifestly differing from each other as fire and water.

R. S.

*London; May* 1820.

---

FOR THE LONDON MEDICAL AND PHYSICAL JOURNAL.

